# Formulation and Evaluation of Transdermal Patches Containing BGP-15

**DOI:** 10.3390/pharmaceutics16010036

**Published:** 2023-12-27

**Authors:** Ildikó Bácskay, Zsolt Hosszú, István Budai, Zoltán Ujhelyi, Pálma Fehér, Dóra Kósa, Ádám Haimhoffer, Ágota Pető

**Affiliations:** 1 Department of Pharmaceutical Technology, Faculty of Pharmacy, University of Debrecen, Nagyerdei körút 98, 4032 Debrecen, Hungary; 2 Institute of Healthcare Industry, University of Debrecen, Nagyerdei körút 98, 4032 Debrecen, Hungary; 3 Faculty of Engineering, University of Debrecen, Ótemető Utca 2-4, 4028 Debrecen, Hungary

**Keywords:** BGP-15, Box–Behnken design of experiment, penetration enhancers, bioavailability, transdermal patches, PVA, PVP, in vitro permeation, porcine skin

## Abstract

BGP-15 is an active ingredient with many advantages, e.g., beneficial cardiovascular and anti-inflammatory effects. The transdermal administration of BGP-15 has great potential, which has not been investigated yet, despite the fact that it is a non-invasive and safe form of treatment. The aim of our study was to formulate transdermal patches containing BGP-15 and optimize the production with the Box–Behnken design of experiment. The most optimal formulation was further combined with penetration enhancers to improve bioavailability of the active ingredient, and the in vitro drug release and in vitro permeation of BGP-15 from the patches were investigated. FTIR spectra of BGP-15, the formulations and the components were also studied. The most optimal formulation based on the tested parameters was dried for 24 h, with 67% polyvinyl alcohol (PVA) content and low ethanol content. The selected penetration enhancer excipients were not cytotoxic on HaCaT cells. The FTIR measurements and SEM photography proved the compatibility of the active substance and the vehicle; BGP-15 was present in the polymer matrix in dissolved form. The bioavailability of BGP-15 was most significantly enhanced by the combination of Transcutol and Labrasol. The in vitro permeation study confirmed that the formulated patches successfully enabled the transdermal administration of BGP-15.

## 1. Introduction

In modern pharmacotherapy, there is an increasing demand to develop new pharmaceutical forms that can be safely administered with adequate bioavailability and patient compliance in order to achieve successful therapeutic responses [1]. Transdermal drug delivery (TDD) is an innovative and appealing alternative form of oral and parenteral drug administration as it uses the skin as its drug-absorbing medium [2]. Although several types of transdermal therapeutic systems (TTSs) can be distinguished, the most common and frequently used forms are transdermal patches [3,4].

These patches are designed to be applied to the skin while delivering a therapeutically effective dose of one or more active ingredients into the systemic circulation through the layers of the skin [4,5,6]. The most basic and important components of transdermal patches are the polymers as they provide numerous functions such as forming the matrix, controlling the rate of drug delivery, providing protection, adhesion, flexibility and permeation [1,7,8,9].

These matrices can be formulated by the dispersion of an active pharmaceutical ingredient in a solid state or liquid polymer base [1,7]. An ideal polymer should meet some requirements such as proper stability and good compatibility with the chemical agent and the other elements that were used; moreover, they should contribute to a predictable drug release [10]. 

The ingredients incorporated into the formulation, the selected polymers and excipients have a significant influence on the physical properties of the patches, as well as their drug release and applicability. During the development of the composition, the properties of the active substance(s) must be considered as an important influencing factor as well [1,11]. The most commonly used polymers in patches are cellulosic derivates (e.g., hydroxypropyl methylcellulose (HPMC)), polyvinyl pyrrolidone (PVP), PVA, polyacrylates and acrylate derivates, as well as chitosan, but of course there are several other options available [10,11,12].

PVA is a copolymer of vinyl acetate and vinyl alcohol that is widely utilized in pharmaceutical formulations for its biocompatible, non-toxic and hydrophilic properties [13,14,15]. The extensive use of PVA is dictated by its beneficial properties, such as outstanding film-forming capability, impressive adhesive and emulsifying characteristics, chemical resistance and mechanical stability. However, PVA-based formulations present some shortcomings as well, which may restrict their use, e.g., insufficient lubrication properties in hydrogels because of the strong action of the intermolecular hydrogen bonds. To overcome these difficulties, some methods have been introduced, among which mixing with PVP showed quite promising results. PVP is a synthetic polymer of N-vinyl pyrrolidone and can be used in the formulation of several drug delivery forms including transdermal patches. PVP has advantageous properties like water solubility, biocompatibility, non-toxicity, inertness, biodegradability and inherent matrix-forming characteristics. Adding PVP to PVA can improve the surface lubrication, and synergistic effects were observed on the structure via the combination of the two polymers [16,17]. The combination of the two polymers has many advantages. However, their ideal ratio differs for each composition and dosage form, thus, it is important to determine the optimal manufacturing conditions and the ratio of solvents and polymers, as these have a significant impact on the physical properties and drug release as well.

In transdermal formulations, sufficient drug penetration is a critical factor. Carefully selected penetration enhancer excipients can support this factor [18,19], and they are able to alter bioavailability and drug penetration as well. Excipients should be selected according to their ability to permeate into different skin layers, considering toxicity and compatibility with all the other components of the formulation [20,21]. There are widespread penetration enhancer excipients and combinations thereof which are preferentially used in external formulations because they can enhance the effect of the active substance by ensuring good drug delivery through the skin [22].

BGP-15 is an active substance currently in the clinical trial stage. It is a solid, yellowish material. BGP-15 is a small molecule, and its molar weight is 351.272 g/mol. Its water solubility is 28 mg/mL at room temperature. BGP-15 is usually administered orally, in the form of capsules. However, the topical administration of the drug also has advantages; in some studies, ointments were formulated as well. Recently, numerous beneficial pharmacological effects of the drug were studied [23]. It is highly effective in the prevention and treatment of diabetes type 2 due to its insulin sensitizing effect [24]. Moreover, BGP-15 has beneficial cardioprotective effects; it is proven to be effective in the treatment of heart failure [25]. In the last few years, the topical administration of the drug was investigated as well. In those studies, photoprotective and anti-inflammatory effects were observed [26,27]. The mechanism of the effect is still not completely understood, but some of the key mechanisms have been identified. BGP-15 is a PARP inhibitor. It increases heat shock protein synthesis, and it is able to increase membrane fluidity as well.

The purpose of our study was to formulate transdermal patches containing BGP-15 as the transdermal administration of the active substance has never been studied before in spite of its great potential in the abovementioned therapeutic indications. In the formulation process, our aim was to prepare PVA- and PVP-based transdermal patches and determine the most suitable proportion of these two polymers, the solvent proportion and the drying time to optimize the process with the help of the Box–Behnken design of experiment. After finding the most optimal composition, our aim was to combine it with different penetration enhancer excipients to improve the bioavailability of BGP-15 and investigate in vitro drug release and the modified permeation test from the patches.

## 2. Materials and Methods

### 2.1. Materials

Transcutol and Labrasol were kind gifts from Gattefossé (Lyon, France). 3-(4,5-Dimethylthiazol-2-yl)-2,5-diphenyltetrazolium bromide (MTT paint), Dulbecco’s modified Eagle’s medium (DMEM), phosphate-buffered saline (PBS), trypsin–EDTA, heat-inactivated fetal bovine serum (FBS), L-glutamine, non-essential amino acids solution, penicillin–streptomycin, polyethylene glycol 400 (PEG 400), PVA and PVP were purchased from Sigma Aldrich. Twelve-well plates were purchased from Corning (Corning, NY, USA). HaCaT cells were supplied from Cell Lines Service (CLS, Heidelberg, Germany). BGP-15 was purchased from SONEAS Chemicals Ltd. (formerly known as Ubichem Pharma Services), Budapest, Hungary. Propylene glycol was obtained from Hungaropharma Ltd. (Budapest, Hungary). 

### 2.2. Formulation of Transdermal Patches

**Figure 1:**
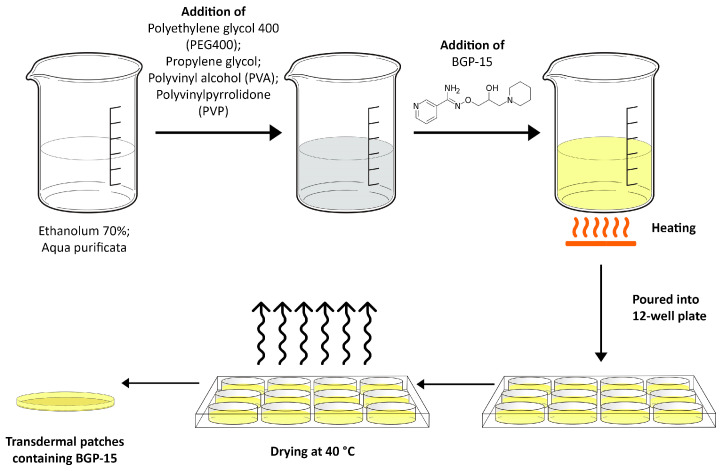
Formulation process of the transdermal patches via the solvent casting method. The polymers (PVA, PVP) and the plasticizers (PEG 400 and propylene glycol) were measured into the mixture of ethanol and purified water. In the next step, the active substance was incorporated into the composition, and then this mixture was heated until we obtained a viscous but clear solution, which was poured into a 12-well plate and dried at 40 °C.

Drug-loaded transdermal patches of BGP-15 were formulated by using the solvent casting method [28]. For this purpose, 12-well plates (diameter 2 cm) were chosen. Polymers (PVA and PVP) were weighed accurately and dissolved in 10 mL of the mixture of water and in ethanol solution of various percentages by volume, diluted from 70% ethyl alcohol and put aside to form a clear solution. The active ingredient (10 *w*/*w*%) was dissolved in the abovementioned solution and mixed until a clear solution was obtained. PEG 400 and propylene glycol were used as plasticizers. The resulting homogeneous solution was cast in the plates and dried at 40 °C for predetermined time intervals. The dried patches were further studied from different aspects. The steps of the formulation are presented in Figure 1.

### 2.3. Design of Experiment

Production of the patches was optimized by using a Box–Behnken design of experiment. During the process, 3 independent and 3 dependent factors were evaluated. Independent variables were the amount of PVA (*w*/*v*%), ethanol content (*w*/*w*%) and drying time (h); these were considered the critical parameters in the production process with an effect on product quality. The experimental factors were varied in the design, at 3 levels in 15 runs/compositions, which are presented in Table 1. The design of experiments considered the reproducibility and the significant effect of each factor on the observed variables. Tensile strength, moisture content and moisture uptake were taken as dependent variables. The quadratic response surface was used to construct a second-order polynomial model using TIBCO Statistica^®^ 13.4 (StatSoft Hungary, Budapest, Hungary). The 3D response surface plots for dependent factors were plotted according to the regression model by keeping one variable at the center level [29].

Preformulation studies were carried out to establish the criteria for the optimization, and the relevant scientific literature was also studied. PVA content of the patches under 30% proved to be extremely sticky, rupturing too easily. Over 70% PVA patches were rigid and difficult to tear, with little elasticity. Scientific literature suggests that PVA should be in the majority when using a mixture of PVA and PVP [17]. Drying time was determined by preformulation studies. Under 12 h, the patches had too high moisture content, that made them too sticky and ruptured too easily, while over 36 h, the patches were overdried, the moisture content was too low, and the patches could not be removed from the plate, so these values were taken as the extreme values during production. Ethanol content provides sufficient solubility of PVA and PVP in the range of 20–50%.

#### 2.3.1. Tensile Strength

Tensile strength and tear characteristics of the polymer films were evaluated using a Brookfield CT3 texture analyzer instrument. It contained two load cell grips, of which the lower one was secured and the upper one was mobile. Film strips with dimensions of 1 × 1 cm were fixed between the cell grips, and force was increasingly applied until the film broke. Tensile strength was measured in mN [30].

#### 2.3.2. Moisture Content

Moisture content of the patches was investigated with a KERN DAB moisture analyzer instrument. The initial weights of the patches were accurately weighed, and they were heated by the instrument to 120 °C. Water content was measured with the instrument, and the moisture content of the films was calculated by taking into account the initial weights and expressed as a percentage [31]:

(1) $$Moisture\;content=\frac{Water\;content}{Initial\;weight}\times 100$$

#### 2.3.3. Moisture Uptake

Moisture uptake of the patches was studied with the help of a climate chamber. The patches were kept in the chamber at room temperature and 80% humidity for 48 h. The initial weights of the patches were previously weighed, and after 48 h they were weighed again [32]. Moisture uptake was calculated based on the weight increase and expressed as a percentage according to the following equation:

(2) $$Moisture\;uptake=\frac{Final\;weight-Initial\;weight}{Initial\;weight}\times 100$$

### 2.4. Penetration Enhancement

After finding the most optimal formulation, composition 4 was further combined with penetration enhancer excipients to improve the bioavailability of BGP-15. The selected excipients were Transcutol and Labrasol, following the suggestions of Gattefossé. Labrasol or Transcutol or a 1:1 mixture of the two excipients was added to the patches at 0.1% during the formulation process.

### 2.5. MTT Assay

To assess the toxicity of the selected excipients, an MTT assay was performed. This procedure was carried out on the HaCaT cell line. The cells were maintained by weekly passages in Dulbecco’s DMEM culture media. For the assay, the keratinocyte cells were seeded on a 96-well plate with the density of 10.000 cells/well. When the cells had completely grown over the well’s membrane, the experiment was ready to perform. First, culture media was removed, then the samples were applied, and the cells were incubated with them for 30 min. After half an hour, the test solutions were removed, and the MTT paint solution was added at a 5 mg/mL concentration to the cells. Then, a 3 h long incubation followed. The viable cells transformed the water-soluble tetrazolium–bromide into formazan precipitate. When the incubation was finished, formazan precipitate was dissolved with the help of isopropanol/hydrochloride acid at a 25:1 proportion. After that, the absorbance of the solutions was measured using a spectrophotometer (Fluostar Optima), and it was directly proportional to the number of viable cells [33]. 

### 2.6. Fourier-Transform Infrared Spectroscopy (FTIR)

The infrared spectra of:

- The active substance (solid BGP-15);
- Transdermal patches with BGP-15, without penetration enhancers;
- The transdermal patches with BGP-15 and the penetration enhancers (Labrasol or Transcutol or the mixture of Labrasol and Transcutol);
- Transdermal patches without penetration enhancers and BGP-15 were obtained by using a JASCO FT/IR-4600 spectrometer with an ATR accessory (Zn/Se ATR PRO ONE Single-Reflection, ABL&E-JASCO, Hungary, Budapest). All the samples were directly placed on the diamond crystal of the equipment. Scanning was run in the wavelength range of 500–4000 cm^−1^. The spectra were collected from 32 scans to obtain smooth spectra at the spectral resolution of 1 cm^−1^. Corrections of environmental CO_2_ and H_2_O were carried out using the software’s built-in method [34].

### 2.7. Scanning Electron Microscopy (SEM)

The morphology of the patches was studied with a Hitachi tabletop microscope (TM3030 Plus, Hitachi High-Technologies Corporation, Tokyo, Japan) in high-resolution mode. The samples were attached to a fixture with a double-sided adhesive tape containing graphite. A low accelerating voltage (15 kV) and vacuum were used to investigate the structure of the cut patches at 500× magnification [13].

### 2.8. In Vitro Release of the Active Ingredient

In vitro drug release was investigated using Franz diffusion chamber apparatus. During the experiment, an artificial cellulose–acetate membrane was placed between the donor and the acceptor phase. Samples were taken from the acceptor phase at predetermined times. The patches were placed on the membrane (0.45 µm pore size) as the donor phase, and as the acceptor phase, a pH = 5.5 buffer was chosen to imitate the skin’s pH with 7 mL/cell volume. The surface area of the cell was 1766 cm^2^. The solubility of BGP-15 is 28 mg/mL in this media [23], thus, theoretically it would be able to dissolve 196 mg BGP-15. The transdermal patches contained 50 mg BGP-15, so the media could dissolve more than triple the amount of the active substance. Prior to the experiment, the membrane was impregnated with isopropyl myristate to match the lipophilic character of the skin. The acceptor phase was set to 32 °C to imitate the temperature of the skin [35].

The diffused amount of BGP-15 was measured with the following HPLC method [36]. The samples were filtered on 0.2 µm polyethersulfone membrane. The sample solutions were analyzed using a HPLC system (Merck-Hitachi, Tokyo, Japan ELITE with photodiode array detector (DAD)). The column was an Aquasil C18 (5 µm 100 × 2.1 mm) with a C18 guard column (5 µm, 4 × 3 mm) and kept at 40 °C, and the DAD was set to 254 nm. The mobile phase was an acetonitrile and water solution at a ratio of 1:9 (containing 0.1% acetic acid), and a 1.0 mL flow rate was used. The analyses were performed with EZChrom Elite software^TM^ 3.2.0. (Hitachi, Tokyo, Japan), which was also used for collecting and processing the data. Standard solution (10 µL) and purified samples were injected.

Dissolution profiles were characterized in several ways. *Flux* was calculated using the following equation:

(3) $$Flux=\frac{Q}{t}$$

where *Q* is the amount of drug released per unit area (mg/cm^2^) and diffusion time (*t*). 

Dissolution curves were fitted to the zero-order, first-order, Korsmeyer–Peppas, Higuchi and Weibull models using a graphical technique.

In order to compare the release values of BGP-15 transdermal patches, difference factors were calculated via a model-independent approach [37]:

(4) $$f_{1}=\frac{\sum_{j=1}^{n}\left|R_{j}-T_{j}\right|\;\;}{\sum_{j=1}^{n}R_{j}}\times 100$$

here, *n* is the sampling number, and *R_j_* and *T_j_* are the percent dissolved of the reference and the test products at each time point *j*, respectively.

(5) $$f_{2}=50\times \log\left\{\;{\left[1+\left(1/n\right)\sum_{j=1}^{n}w_{j}{\left|R_{j}-T_{j}\right|}^{2}\;\right]}^{-0.5}\times 100\;\right\}$$

here, *w_j_* is an optional weight factor.

### 2.9. In Vitro Permeation Studies

In vitro permeation studies were performed in Franz diffusion cell apparatus with a modification whereby the artificial cellulose acetate membrane was replaced by previously prepared pieces of pig ear skin. As acceptor phase, pH = 5.5 phosphate buffer was chosen, with 7 mL/cell volume. The pig ear was obtained from a slaughterhouse. Skin slices of 1 mm thickness were isolated from the inner, thinner, intact side of pig ears and were then frozen at −18 °C until the experiment. The isolated skin samples were defrosted and rehydrated in physiological saline solution with sodium azide (0.01 *w*/*v*%) at 25 °C for 30 min to preserve functionality. After washing with azide-free saline solution, the skin layer was used instead of the membrane used in the in vitro experiment (Section 2.8, In Vitro Release of the Active Ingredient). The stratum corneum faced upwards in the donor phase [38].

### 2.10. Determination of BGP-15 in Skin Samples

The amounts of BGP-15 that accumulated in the skin and in the acceptor phase were determined at the end of the in vitro permeation studies. The used porcine skin underwent solvent extraction to determine the distribution of BGP-15 between the stratum corneum and the dermis with epidermis. At the end of the permeation test, the application surface of the ear skin was washed with physiological washing saline solution to remove the sticky formulation. Then, the skin was dried with the help of cotton wool, and adhesive cellophane tapes were used to remove the stratum corneum by tape-stripping 25 times. The BGP-15 content was washed from the stripping tapes with 5 mL of methanol into glass vials. The remaining stripped skin (epidermis without stratum corneum plus dermis) was then cut into small pieces that were placed in 5 mL of methanol for solvent extraction. Extractions were carried out in an ultrasonic bath at 40 °C for 15 min and repeated three times. The samples were then evaporated under a stream of N_2_ gas at 45 °C and dissolved back into the mobile phase with the help of an ultrasonic bath (5 min). The samples were then centrifuged (4000 rpm, 15 min), and the supernatants were filtered in a 0.2 µm PES membrane [38]. 

### 2.11. Statistical Analysis

Data were analyzed with GraphPad Prism 6 and presented as means ± SD. Comparison of the groups in MTT assays was performed with one-way ANOVA test and the *t*-test. Significant differences in the figures are indicated with asterisks. Differences were regarded as significant at *p* < 0.05. All experiments were performed at least in triplicate.

## 3. Results

### 3.1. Design of Experiment

#### 3.1.1. Tensile Strength

**Figure 2:**
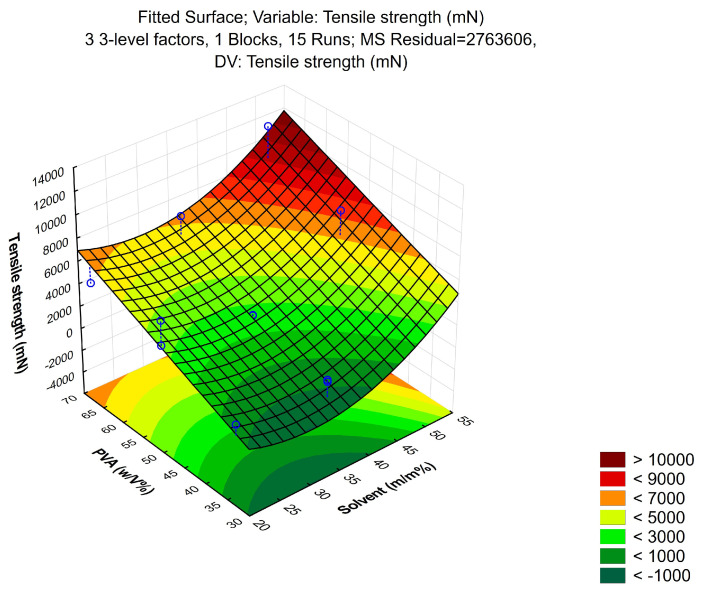
Increasing the concentration of PVA in the composition increased the tensile strength of the transdermal patches. As for the solvent, ethanol in 20–40 *w*/*w*% concentration slowly increased the tensile strength, while the further increase in ethanol content resulted in a faster ascending section.

The drying time did not show any significant difference in the tensile strength, therefore, only the correlation between PVA and the solvent was investigated. The curve in Figure 2 shows that increasing the concentration of PVA increased the tensile strength of the patch linearly, and the 3D surface displays that PVA rundown was linear. As for the solvent, a non-linear correlation was observed, and the plot could be well described by an exponential trend. At 20–40 *w*/*w*%, a slower increase in the tensile strength was observed, while further increasing the ethanol concentration in the solvent mixture resulted in a faster ascending section. 

#### 3.1.2. Moisture Content

**Figure 3:**
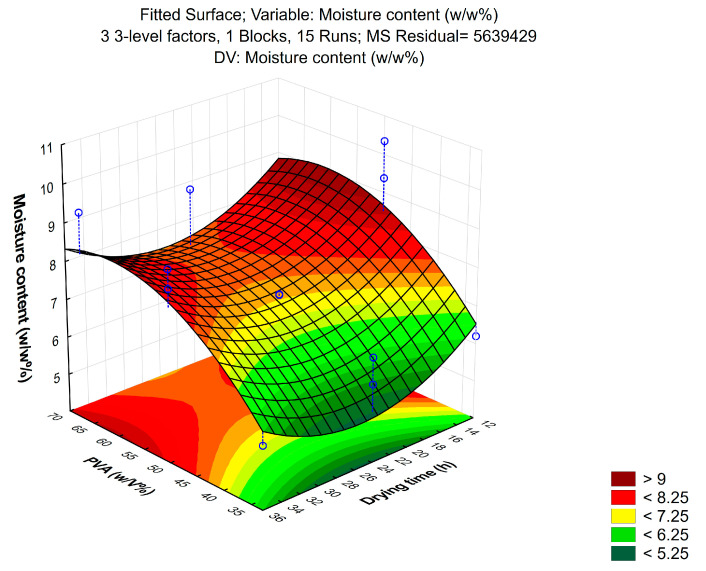
The correlation of PVA concentration and moisture content shows that increasing PVA content resulted in an increase in moisture content, while the increase in drying time decreased moisture content.

The moisture content of the formulated patches was not significantly affected by the solvent; however, PVA concentration and drying time significantly influenced it. Figure 3 represents the results. Increasing the concentration of PVA resulted in an increase in the moisture content, which no longer increased linearly after a certain polymer concentration. Increasing the drying time decreased the moisture content up to 24–30 h; afterwards, there was no significant difference in the curve.

#### 3.1.3. Moisture Uptake

Moisture uptake was not affected by the investigated factors (PVA concentration, ethanol concentration and drying time) during the formulation. No significant differences were detected. The average moisture uptake of the patches was 22.05 ± 4.06 *w*/*w*%.

#### 3.1.4. Incorporating Penetration Enhancers into the Formulation

Based on the result of the Box–Behnken design of experiment, composition 4 was proved to be the most optimal formulation with 67% PVA, 20% ethanol content and 24 h of drying time. Thus, it was further combined with penetration enhancer excipients to improve the bioavailability of BGP-15. Transcutol and Labrasol were chosen as the penetration enhancers. The formulations are listed in Table 2.

### 3.2. MTT Assay

**Figure 4:**
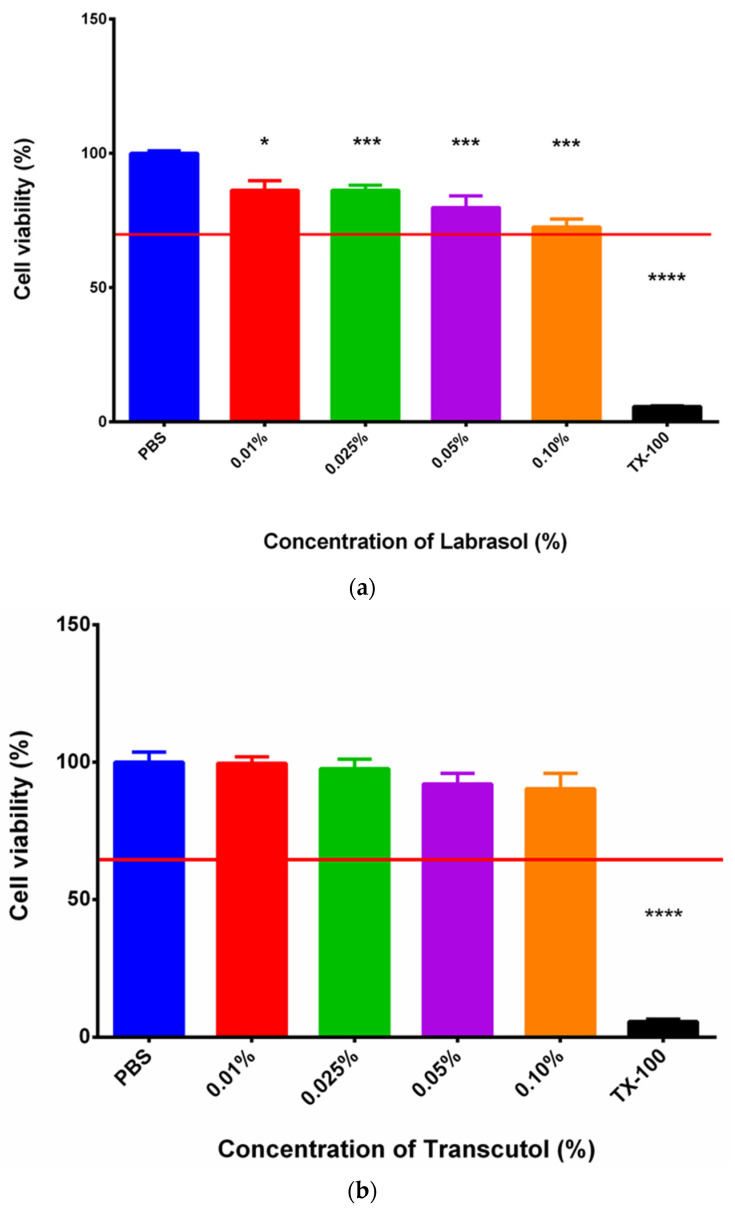
Cytotoxicity of Labrasol (**a**); cytotoxicity of Transcutol (**b**). Cell viability was determined as the percentage of PBS. The concentrations used in the formulations proved to be safe in all cases, and cell viability values were above 70% (marked with a red line) in every case. Data represent the mean of 6 wells ± SD. For statistical analysis, the *t*-test and one-way ANOVA test were carried out. Significant difference is marked with asterisks. *, *** and **** show statistically significant difference at *p* < 0.05; *p* < 0.001 and *p* < 0.0001.

The results of the MTT assays are represented in Figure 4a,b. The two penetration enhancer excipients, Labrasol and Transcutol, were dissolved in PBS prior to the experiment, and different concentrations of these materials were tested on HaCaT cells. PBS was selected as a negative control and Triton-X 100 as a positive control. The values of cell viability were compared with those in PBS, and they are indicated as a percentage. In the experiment, both Labrasol and Transcutol proved to be safe and well tolerated by the cells in the tested concentration range, since cell viability values were above 70%.

### 3.3. FTIR Measurements

**Figure 5:**
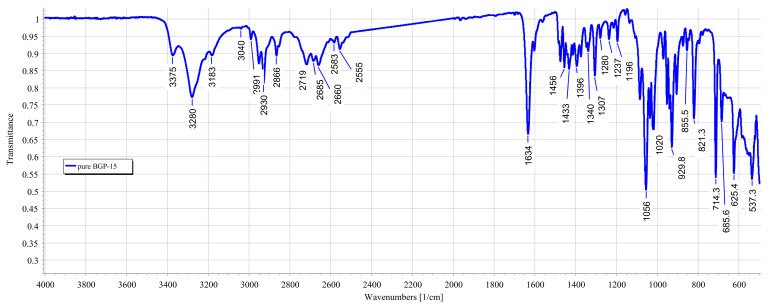
FTIR spectrum of BGP-15. A broad region in the 3200–3400 range was observed, characteristic of the OH group. The characteristic peaks of the amine group were 3375, 3279 and 1634 cm^−1^, respectively. Characteristic peaks of the ether group were found at 2991 and 2929 cm^−1^.

The characteristic chemical groups of BGP-15 (N′-[2-hydroxy-3-(1-piperidinyl) propoxy]pyridine-3-carboximidamide) were studied using the FTIR method. The IR spectra showed the location of the characteristic groups. A broad region in the 3200–3400 range was observed, characteristic of the OH group. The characteristic peaks for the amine group were 3375, 3279 and 1634 cm^−1^, respectively. Characteristic peaks for the ether group were found at 2991 and 2929 cm^−1^. The FTIR spectrum of BGP-15 is presented in Figure 5.

**Figure 6:**
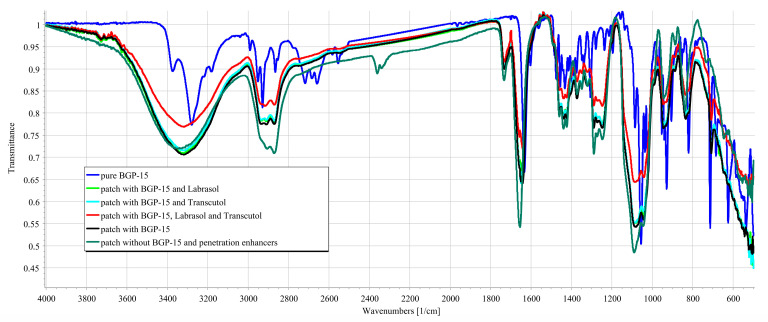
FTIR-spectra of the active substance (solid BGP-15), transdermal patches with BGP-15, without penetration enhancers, the transdermal patches with BGP-15 and the penetration enhancers (Labrasol or Transcutol, or the mixture of Labrasol and Transcutol), transdermal patches without penetration enhancers and BGP-15.

During the formulation of the patches, BGP-15 formed secondary bonds with the polymers used as the base for the patch. This was indicated by the band shift of BGP-15 amine groups from 1634 to 1641. No further band shifts were found when penetration enhancers were incorporated into the composition. The results confirmed that BGP-15 was in a soluble form in the formulation and did not degrade chemically during the preparation. FTIR spectra are summarized in Figure 6.

### 3.4. SEM 

**Figure 7:**
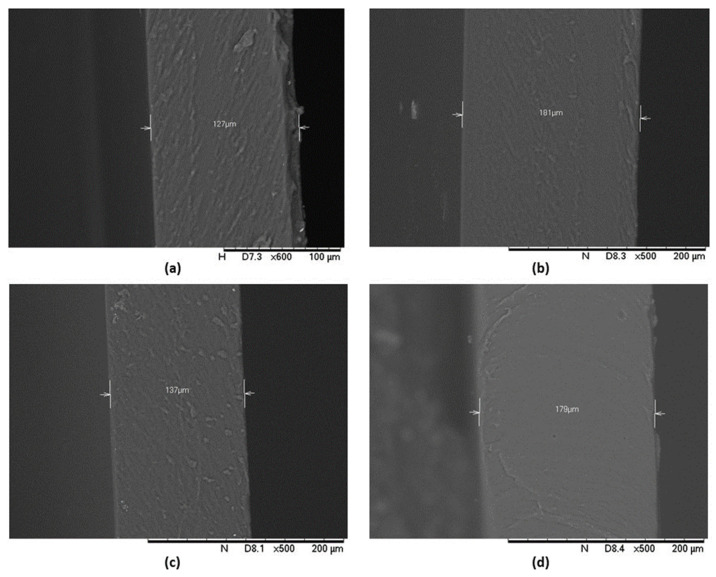
SEM photography of BGP-15 containing transdermal patches without penetration enhancers (**a**), with Labrasol (**b**), with Transcutol (**c**) and with the combination of Labrasol and Transcutol (**d**).

The morphology of the patches was studied via scanning electron microscopy. The photographs confirmed the compatibility of BGP-15 with the polymer matrix as it showed homogeneous films. The thickness of the patches was measured as well; it was between 120 and 190 µm. The scans of the transdermal patches are demonstrated in Figure 7.

### 3.5. In Vitro Drug Release

**Figure 8:**
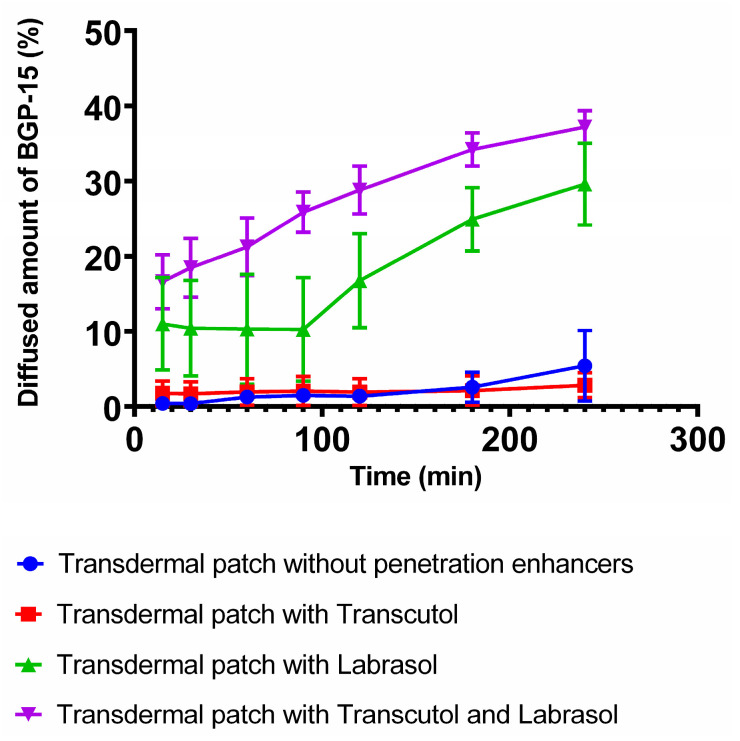
Release profiles of BGP-15 across the cellulose acetate membrane from the transdermal patches. The best result was obtained by the formulation which contained the combination of Transcutol and Labrasol, while the lowest release values belonged to the patches containing Transcutol or no penetration enhancer excipient at all.

Figure 8 represents the diffused amount of BGP-15 across the isopropyl myristate-impregnated cellulose acetate membrane from the different transdermal patches with or without penetration enhancers. Those compositions, which contained Labrasol or the combination of Labrasol and Transcutol achieved higher drug release values. The best result was achieved by using the combination of Transcutol and Labrasol, where the diffused drug amount was 37.19%. The second-best result was obtained by the patch formulated with Labrasol, where the diffused drug amount was 29.58%. The lowest release rates were achieved by the patches prepared with Transcutol or without any penetration enhancer excipient.

Comparing the flux of the compositions (Table 3), the patch with Transcutol and Labrasol showed a nearly tenfold higher value than the patch without enhancers. The Transcutol sample alone showed no significant difference from the composition without enhancers.

Kinetic model fitting showed the difference between the formulations in Table 4. The Weibull model best described the drug release of patches without penetration enhancers. In the case of penetration enhancers, drug release was better described by different modeling. Transcutol showed zero-order kinetics, while Labrasol showed first-order kinetics. When the penetration enhancers were used together, first-order kinetics showed the best correlation.

Release profiles of the transdermal formulations were compared. The calculated similarity and difference factors are listed in Table 5. Two formulations were recognized as similar if their similarity factor (*f*_2_) was between 50 and 100, and different if their difference factor (*f*_1_) was between 0 and 15. According to the calculated values, a great similarity was confirmed between the patches, which were formulated without penetration enhancers or with Transcutol. Transdermal patches prepared without penetration enhancers or Labrasol were significantly different. Patches without penetration enhancers compared with the combination of Transcutol and Labrasol turned out to be significantly different, as did the Labrasol-containing patch versus the combined one.

### 3.6. In Vitro Permeation

**Figure 9:**
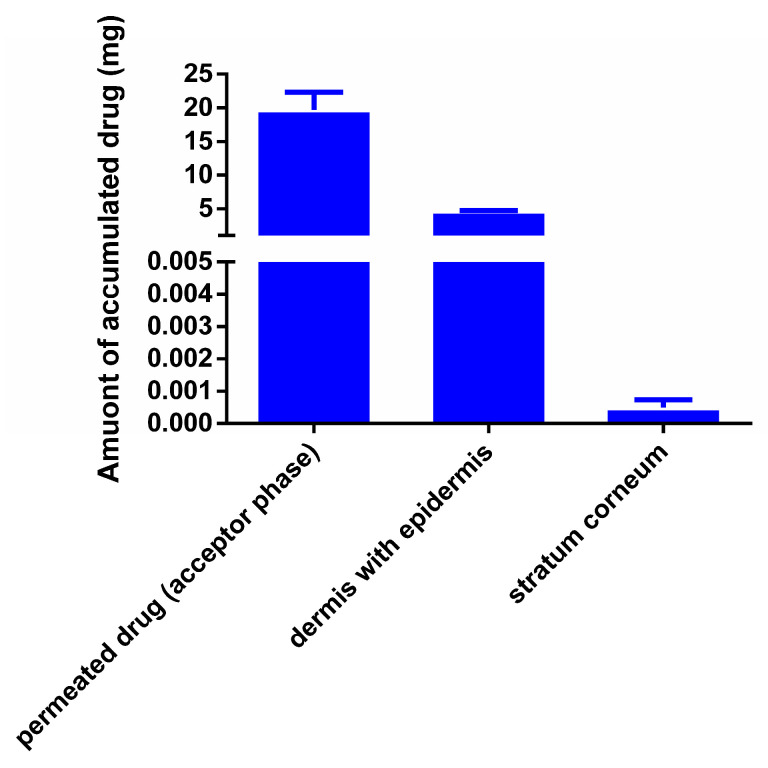
In vitro permeation of BGP-15 across porcine skin; 39.3% (19.69 mg) of the initial BGP-15 quantity was able to permeate to the acceptor phase.

In Figure 9, the results of the in vitro permeation study are presented. In this experiment, the permeation of the patch formulated with the combination of Transcutol and Labrasol was studied across pig’s ear skin. According to the results, 39.3% (19.69 mg) of BGP-15 was able to permeate to the acceptor phase through the skin. In the skin (dermis plus epidermis), 9.2% of the initial BGP-15 was detected. In the case of skin, the flux of BGP-15 was 0.4647 mg/cm^2^ × h^−1^.

## 4. Discussion

BGP-15 has been intensively studied in recent years, and many favorable effects of the drug candidate have been identified throughout this period. Beside the insulin sensitizing effect [39], advantageous cardioprotective [40,41] and anti-inflammatory effects [36] were observed. Transdermal administration is an under-investigated research area of BGP-15, as is topical application, since few studies on its utilization in ointment form are available. In our previous work, we investigated the topical application and the anti-inflammatory effect of the active ingredient [36]. The possibility of applying BGP-15 transdermally counts as a novelty as it has never been investigated before, although it could be particularly advantageous in the abovementioned indications. 

In the present study, transdermal patches were formulated to promote the transdermal delivery of the active substance. Some of the most commonly used polymers in transdermal patches are PVA and PVP. The combination of these two polymers can be especially beneficial. However, very little information is available in the scientific literature about their ideal proportion in transdermal patches, the proper solvents and the circumstances of an optimal production. In our series of experiments, we aimed to determine the optimal production conditions of transdermal patches with the two polymers PVA and PVP to ensure the suitable transdermal dosage form for BGP-15. To optimize the production, the Box–Behnken design of experiment was used. In the factorial design, PVA content proved to be a key factor, as it increased the tensile strength and the moisture uptake as well, which are important characteristics of transdermal patches. The ethanol content was also important; at a 20–40% concentration it resulted in an increase in tensile strength. Based on these results, composition 4 was selected as the most optimal formulation with 67% PVA, 20% ethanol content and 24 h of drying time. The PVA content of the transdermal patches has key importance, as it determines the tensile strength. Lee et al. studied different transdermal patches and found that the larger the PVP proportion was in the formulation, the worse mechanical strength the composition had [17]. This complies with our experiences and results, as a PVP content above 50% resulted in patches that tore too easily. Gao et al. formulated transdermal patches with a PVA and PVP base. They used different ratios of polymers. They found that the larger PVA proportion worked the best; according to their results, a 7:1 PVA/PVP proportion was the most ideal as it determined the drug release and the quality of the transdermal patches [42]. The alcohol content influences the polymer expansion and the formation of secondary interactions [43]. Due to the solubilities of PVA and PVP [44], it is important to determine the ideal alcohol content where both molecules can unfold and interact to form a stable matrix after drying, which serves as the basis for our patch. Due to the PVA or PVP content of the films, the drying time at a given temperature is an important parameter. The drying time affects the recrystallisation of the polymers and the physical changes in their structure. As drying time increases, the hydration decreases, which can lead to loss of secondary interactions and crystallization [45].

To increase the bioavailability of BGP-15, different penetration enhancer excipients were incorporated into composition 4, namely, 0.1% Transcutol or 0.1% Labrasol or the combination of these two at 0.1%. Since safety is an important aspect of pharmaceutical formulations, cytotoxicity of these excipients was investigated using MTT assay on the HaCaT cell line. Based on the results, the selected excipients in the used concentration were safe and well tolerated, and cell viability was above 70%, which is the recommendation of ISO 10993-5 [46]. 

FTIR spectra of BGP-15 were first described by our research group. Besides BGP-15 itself, all the transdermal patches were studied using the FTIR method. The results confirmed the compatibility of the vehicle and the active substance; BGP-15 was dissolved in the polymer matrix. Taher et al. studied isonicotinic acid in PVA blend, and according to their FTIR measurements, the active substance was in dissolved form, similar to our case [47]. The results of SEM confirmed this as well since no crystals or other particles were found in the SEM photography. This also confirmed the theory that PVP inhibits the crystallization of substances.

In vitro drug release from the patches was investigated using Franz diffusion chamber apparatus. The active ingredient is present in a dissolved form, so penetration was expected to be faster than in crystalline form, which was confirmed by the in vitro drug release test. In this study, the best results were achieved by those formulations which contained both penetration enhancers Labrasol and Transcutol in combination. The second-best results were obtained by the Labrasol-containing patches, and those which contained Transcutol or no penetration enhancer at all achieved lower release values. Even though Transcutol is a well-known and powerful penetration enhancer, in some cases it is not able to enhance drug release and permeation [48]. Labrasol is a widespread and excellent penetration enhancer as well. In combination with Transcutol, the highest drug release was observed in our in vitro experiment, which can be explained by the synergistic penetration enhancer effect of the two excipients. Transcutol is often used in combination with other penetration enhancers; Amitkumar et al. found that oleic acid and Transcutol in combination significantly improved the transdermal drug delivery of oxcarbazepine [49]. The dissolution from the patches without penetration enhancers was well described with the Weibull model. The model is useful for determining the release profiles of matrix-type drug delivery. The correlation was obtained with slightly lower values for the Korsmeyer–Peppas model, which is suitable for predicting the release mechanism from a polymeric system [50]. The Higuchi relationship is used in the case of modified transdermal systems [51], nevertheless, it resulted in the worst match to the dissolution of patches as in other articles [52]. Penetration enhancers have already been described to affect drug delivery differently depending on the solubility or pKa value of the active substance [53,54]. Preparations containing a penetration enhancer can be described better with first- or zero-order kinetics.

The in vitro permeation study was carried out with composition 4 combined with the mixture of Labrasol and Transcutol, as this formulation achieved the best results through the synthetic membrane. A total of 39.3% of the initial BGP-15 was able to permeate to the acceptor phase, and only 9.2% BGP-15 was detected in the skin. This leads to the conclusion that BGP-15 does not accumulate in the skin but rather in the acceptor phase, so the formulated transdermal patch is suitable for transdermal delivery of the active ingredient. BGP-15 was present in a semi-dissociated form at pH 5.5, which facilitated its permeation through biological membranes; thus, a fast and efficient permeation was observed, as expected. Minor BGP-15 accumulation was measured in the skin. Drug flux across the biological membrane was reduced, as expected, due to the more complex system. According to the results, the formulated transdermal patch enables BGP-15 delivery to the systemic circulation; thus, the formulation provides a new therapeutic option and improved patient compliance.

The novelty of our work is the transdermal application of BGP-15, the development of an optimal transdermal dosage form for the active substance and the improvement of bioavailability with penetration enhancers. FTIR spectra of the active substance were characterized for the first time in the scientific literature.

## 5. Conclusions

In our series of experiments, we aimed to complete the currently available knowledge about BGP-15 by developing a suitable transdermal formulation for it and improving its bioavailability with penetration enhancers. Our study highlights the relevance of choosing appropriate excipients as it highly affects the formulation in many aspects. In the research, the ideal proportion of the selected polymers was determined, as well as the solvent ratio and the circumstances of the production. By further combining the most optimal composition, we were able to enhance the bioavailability of BGP-15. In the in vitro tests of drug release and permeation, we confirmed that the formulation is suitable for transdermal delivery of the active substance. This research enables and supports the transdermal administration of BGP-15, which is an important and promising perspective considering its indication field.

## Figures and Tables

**Table 1:** Compositions of the formulated transdermal patches. Each composition was prepared using the solvent casting method and dried for predetermined time intervals.

Composition	Solvent (*w*/*w*%)	PVA (*w*/*v*%)	Drying Time (h)
**1**	50	33	24
**2**	20	33	24
**3**	50	67	24
**4**	20	67	24
**5**	50	50	12
**6**	20	50	12
**7**	50	50	36
**8**	20	50	36
**9**	35	33	12
**10**	35	67	12
**11**	35	33	36
**12**	35	67	36
**13**	35	50	24
**14**	35	50	24
**15**	35	50	24

The solvent was the mixture of purified water and ethanol (70 *v*/*v*%). In the table, the quantity of ethanol is represented in the solvent mixture. The selected polymers for the films were PVA and PVP. In the table, the quantity of PVA is displayed from the mixture of the polymers. The center point formulations are marked with gray color.

**Table 2:** The composition of the transdermal formulations in combination with penetration enhancers.

Formulation	
W.P.	Transdermal patch without any penetration enhancer excipient
T	Transdermal patch with 0.1% Transcutol
L	Transdermal patch with 0.1% Labrasol
T + L	Transdermal patch with 0.1% Transcutol and Labrasol

**Table 3:** Summary of the dissolution kinetic model.

	Transdermal Patches			
	Without Penetration Enhancers	With Transcutol	With Labrasol	With Transcutol and Labrasol
Flux (mg/cm^2^ × h^−1^)	0.3843	0.2007	2.0940	2.6324

**Table 4:** Summary of the dissolution kinetic model.

Kinetic Model ^1^	Transdermal Patches			
	Without Penetration Enhancers	With Transcutol	With Labrasol	With Transcutol and Labrasol
Zero	0.8780	0.8107	0.8977	0.9772
First	0.8741	0.8100	0.8984	0.9857
Korsmeyer–Peppas	0.8871	0.6630	0.6253	0.9636
Higuchi	0.7494	0.7600	0.8668	0.9597
Weibull	0.9097	0.6624	0.6251	0.9578

^1^ The table contains the correlation coefficient value of the fitting line.

**Table 5:** Difference and similarity factors to compare the release profiles of the formulations (W.P.: transdermal patch without penetration enhancer; T.: transdermal patch formulated with Transcutol; L.: transdermal patch formulated with Labrasol; T and L.: transdermal patch formulated with the combination of Transcutol and Labrasol).

Formulation	*f*_1_ ^1^	*f*_2_ ^2^
W.P. vs. T.	9.45	89.48
W.P. vs. L.	88.53	40.39
W.P. vs. T and L.	92.88	30.17
L. vs. T and L.	37.91	49.17

^1^ Two formulations are recognized as similar if their similarity factor (*f*_2_) is between 50 and 100. ^2^ Two formulations are recognized as similar if their difference factor (*f*_1_) is higher than 15.

## Data Availability

Data are available from the corresponding author with the permission of the head of the department. The data that support the findings of this study are available from the corresponding author (peto.agota@pharm.unideb.hu) upon reasonable request.
